# Oligonucleotides targeting coagulation factor mRNAs: use in thrombosis and hemophilia research and therapy

**DOI:** 10.1186/s12959-017-0130-8

**Published:** 2017-03-07

**Authors:** Marco Heestermans, Bart J.M. van Vlijmen

**Affiliations:** 1 Einthoven Laboratory for Experimental Vascular Medicine, Leiden University Medical Center, Leiden, The Netherlands; 2 Department of Internal Medicine, Division of Thrombosis and Hemostasis, Leiden University Medical Center, Leiden, The Netherlands

**Keywords:** siRNA, Antisense oligonucleotides, RNA, Therapy, Liver, Coagulation

## Abstract

Small interfering (si) RNAs and antisense oligonucleotides (ASOs; here for simplicity reasons, both referred to as oligonucleotides) are small synthetic RNA or DNA molecules with a sequence complementary to a (pre)mRNA. Although the basic mechanisms of action between siRNAs and ASO are distinct, a sequence-specific interaction of the both oligonucleotides with the target (pre)mRNA alters the target’s fate, which includes highly effective sequence-specific blockade of translation and consequently depletion of the corresponding protein. For a number of years, these oligonucleotides have been used as a tool in biological research to study gene function in vitro. More recently, safe and specific delivery of these oligonucleotides to the liver of mammals has been achieved and optimized. This not only allowed their use for in vivo gene studies in physiology and disease, but also opened the opportunity for the development of a new generation of RNA-specific drugs for therapeutic purposes. In 2013, the first oligonucleotide product targeting RNA from the hepatic cholesterol pathway was approved. For blood coagulation, a large portion of key proteins are produced in the liver, and thereby siRNAs and ASOs can also be used as appropriate tools to target these proteins in vivo. In this review, we describe the first use of oligonucleotides for this purpose from zebrafish to primates. As the use of oligonucleotides allows avoidance of early lethality associated with full deficiency of several coagulation factors, it has proved to be of value for studying these proteins in physiology and disease. Currently, oligonucleotides are tested as therapeutics, with the ultimate goal to beneficially modulate the hemostatic balance in thrombosis and hemophilia patients. We discuss both the preclinical and clinical studies of a number of siRNAs and ASOs with the potential to be introduced as drugs for prophylactic and/or treatment of thrombosis or hemophilia. We conclude that for the coagulation field, oligonucleotides are of value for research purposes, and now the moment has come to fulfill their promise as therapeutics.

## Background

Small interfering (si) RNAs and antisense oligonucleotides (ASOs; here for simplicity reasons, both referred to as oligonucleotides) are small synthetic RNA or DNA molecules with a sequence complementary to a (pre)mRNA. Sequence-specific interaction of the siRNA or ASO with the target (pre)mRNA alters the target’s fate, which includes sequence-specific blockade of translation and consequently depletion of the corresponding protein. siRNAs and ASOs have potential in research and therapy; design of sequence-specific oligonucleotides and testing their efficacy in vitro can be relatively easily achieved, and off-target effects are limited. Moreover, siRNAs and ASOs have a relatively simple chemistry, malleability, and delivery (at least in vitro), allowing the oligonucleotides to become a frequently used tool in biological research for studying gene function in a variety of eukaryotic cell types. The major differences between siRNAs and ASOs comprise differences regarding their chemical structure and their mode of action, but considering their primary biological impact i.e., silencing the target (pre)mRNAs target, both are highly effective [1–3].

Already in 1978, it was shown that ASOs proved successful in targeting (pre)mRNAs in vivo in experimental animals [4]. More recently, in 2003, also siRNAs showed strong in vivo potency [5], and delivering siRNAs to the liver proved feasible and successful [6, 7]. siRNAs and ASOs both successfully escape degradation in both the blood circulation and intracellular lysosomes, and effectively reach the appropriate cellular compartment to find their complementary (pre)mRNA. Hepatic delivery allowed the use of oligonucleotides for in vivo gene function studies in normal (hepatic) physiology or for disease analogues to the in vitro approach. Moreover, siRNAs and ASOs allow validation of therapeutic targets in pre-clinical trials forming the stepping stone towards clinical application [8–10]. As blood coagulation factors are predominantly expressed in the liver, venous thrombosis and hemophilia are diseases that may particularly benefit from the oligonucleotide approach; both when it comes to use as a research tool in preclinical research as well as an approach for therapy.

In this review, we will discuss the possibilities for using oligonucleotides in venous thrombosis and hemophilia research and therapy. First, we will outline the chemical and cellular differences between siRNAs and ASOs. Next, we will discuss their usefulness to study coagulation physiology and pathophysiology (i.e., thrombosis and hemophilia) in animal models. Finally, we will examine the current status of the exciting first clinical applications of oligonucleotide approach in venous thrombosis and hemophilia, and the potentials that come along with this novel treatment modality.

## siRNA and ASO biology and delivery

Although the target of siRNAs and ASOs is the same i.e., the (pre)mRNA of the gene of interest, their mechanism of action is different. In short, siRNAs are synthetic RNA duplexes consisting of two unmodified annealed 21-mer oligonucleotides. When siRNAs enter the cytosol, the antisense strand i.e., the strand which specifically targets an mRNA, forms a complex with an endoribonuclease enzyme called Dicer for loading into RNA-induced silencing complex (RISC), which enables their stabilization. A single RISC, with the RNase Argonaute-2 (for which siRNA competes with microRNAs, as discussed in [11]) as its functional unit for accommodating RNA breakdown and the antisense strand for the specificity, can target and break down multiple mRNAs in the cytosol. This process is also known as RNA interference [1, 12]. Whereas siRNAs enter the cell as a duplex, ASOs are single stranded antisense nucleic acids (DNA, RNA, or an chemical analogue), typically 8–50 nucleotides long. ASOs are not recognized by Dicer and not built into RISC upon entering the cytosol; depending on their chemical modification, they directly recognize their (pre)mRNA target in the cytosol or nucleus, due to their complementarity [13]. ASOs can block, break down (after recruitment of RNase H), or induce exon skipping of the mRNA [2]. In contrast to siRNAs, where a single RISC can degrade multiple mRNAs, ASOs typically interact with a single (pre)mRNA.

With the desire to use oligonucleotides in vivo, the delivery to the correct cell and cellular compartment without being degraded has been an important issue. Thus far, the delivery of oligonucleotides to the liver in mammals is an area where significant progress has been made: siRNAs can be packaged in liposomes with a high tropism for the liver, and they can be conjugated to specific ligands (the so called- N-acetylgalactosamine (GalNAC) ligands, which allows the uptake via hepatocyte-specific asialoglycoprotein receptors) which mediate receptor-mediated take-up by hepatocytes [14–17]. A liposome-based formulation and hepatic delivery has also been described for other oligonucleotides [18]. Upon systemic delivery, the biodistribution of ASOs is broad, and the highest concentrations are typically found in the liver and the kidneys [19–21]. This delivery is an aspecific process and works via a yet unknown mechanism, however it is likely that the relative amphipathic nature of molecules contribute to this cell-specific distribution. Differences in the delivery of the compound (packaging versus an aspecific process) and the later discovery of (the functioning of) siRNAs are probably the main reasons why ASOs are ahead for entering into (pre)clinical trials. More recently, it has been reported recently that ASOs can alsobe GalNAc conjugated, and that they are (successfully) tested in (pre)clinical trials [22, 23].

Because the liver appears to be the most successfully targeted organ thus far - although there are interesting reports showing e.g., successful delivery of siRNAs to endothelium and leukocytes [24, 25] - we will focus in this review on targeting mRNA encoded by genes expressed in hepatocytes. This successful hepatic delivery of oligonucleotides makes an mRNA-targeting approach specifically interesting for studies and eventually therapies where a liver-produced protein is a major contributor to the physiology and/or pathophysiology of a certain disease, such as hepatitis, hypercholesterolemia, hemophilia, and thrombosis [26]. For this review, we will focus solely on the use of oligonucleotides for targeting liver-produced coagulation proteins, to study their role in biology and modulate their activity in hemophilia and thrombosis with the ultimate goal to fight or prevent the disease.

## siRNAs and ASOs to study coagulation gene function in physiology and disease

A conventional approach to study gene function in vivo is by generating genetically modified animals. Recently, the developments leading to CRISPR/cas9-mediated genome editing have allowed generation of mutant and/or knockout within a period of months [27]. Nevertheless, extensive breeding programs, also for the generation of proper (littermate) controls, remain expensive, and gene deletion may preclude detailed study of gene function due to perinatal lethality or gene redundancy. Regarding coagulation, most genes from the extrinsic, common, or anticoagulant pathways have been deleted in mice, which often resulted in early lethality, mostly as a result of coagulopathy [28, 29].

As an alternative to the genetic modification approach, oligonucleotides can be used to transiently silence a gene of interest. Using oligonucleotides is simple, fast, cheap, and there are no issues with generating the appropriate controls, because a group of identical mice can be injected with a scrambled control sequence, which is structurally comparable. Moreover, because of the acute silencing of oligonucleotides, analyses can be performed before potential compensatory pathways have become active, and results can be easy translated to other species (including human), as long as the genome has been sequenced.

## siRNAs and ASOs in animal models for coagulation - zebrafish

A relatively new animal model for the study of blood coagulation and related processes is the zebrafish (*Danio rerio*). These fish possess a conserved and sophisticated coagulation system, which in essence follows the same principles as the system in mammals [30, 31]. Moreover, the low cost of maintenance and their low turnover time between subsequent generations make them appealing for (bio)medical research.

The oligonucleotide of choice in this animal are the so-called morpholinos, a subclass of ASOs which got their name because their backbone consists of methylenemorpholine rings [32]. These oligonucleotides do not degrade the targeted mRNA, but rather block translation by making the target mRNA inaccessible for ribosomes. The coagulation factors fibrinogen, prothrombin, and factor VII have been silenced in vivo using morpholinos.

For fibrinogen, zebrafish produce orthologues for the three chains of the mature protein, and it has an in vivo expression pattern comparable to mammals. Moreover, fibrinogen strands are incorporated in the (experimental) thrombus of a zebrafish, and upon morpholino-mediated downregulation of either of the three orthologues, larvae suffer from intracranial and intramuscular hemorrhages [33].

Morpholino-mediated downregulation of prothrombin causes two distinct phenotypes in zebrafish larvae. Some larvae suffer from serious developmental issues causing premature death (even before blood flow has developed), while others develop a bleeding phenotype during a later stage of embryonic development [34]. These results recapitulate the hemophilic phenotype that is seen in experimental mammalian animals with deficiencies of these factors. Moreover, a microarray analysis was performed on mRNA from zebrafish treated which were with prothrombin-morpholinos [35]. A total of 63 upregulated or downregulated genes were identified, although their exact function remains to be determined.

Using coagulation factor VII-specific morpholinos, it was shown in zebrafish that hepsin plays a crucial role in activating factor VII [36]. Besides fibrinogen, prothrombin, and factor VII, more genes involved in hemostasis, such as von Willebrand factor and genes involved in thrombocyte production and function, have been targeted in zebrafish using morpholinos [37–39].

Although translating results from zebrafish to humans remains difficult due to a wide evolutionary gap between both species, the presented data imply that using zebrafish-specific advantages can contribute in unraveling processes of hemostasis, with a clear role for the use of oligonucleotides as a tool.

## siRNAs and ASOs in animal models for coagulation - mammals

In order to study processes involving coagulation factors, mammalian animal models, such as mice, rats, and rabbits, are genetically closer related to humans. In these mammals it has been shown that siRNAs and ASOs can be delivered to the liver in vivo to silence (pre)mRNAs of proteins produced in hepatocytes [26, 40–42]. Several genes involved in the extrinsic, common, or anticoagulant pathway of coagulation have been targeted.

Antithrombin, protein C, and protein S are important natural anticoagulants, and individuals with partial deficiencies in either of these factors have an increased risk to develop venous thrombosis, while full deficiency is rare and very severe or incompatible with life [43]. Genetically modified mice missing either of these anticoagulants die *in utero* because of thrombotic complications. However, using siRNAs, the anticoagulants antithrombin or protein C can be silenced transiently in adult mice to study them in a less anticoagulant environment. When both natural anticoagulants are silenced simultaneously mice develop spontaneous venous thrombosis, a phenotype which is characterized by the formation of fibrin-rich thrombi in the head and rapid consumption of coagulation factors [44].

This mouse model allowed studying factors involved in (experimental) venous thrombosis pathophysiology. Platelets appeared to be crucial for spontaneous venous thrombosis to occur, whereas neutrophils were not rate limiting, and lowering of plasma coagulation factor XII surprisingly seemed to aggravate rather than rescue the thrombotic phenotype [45].

Oligonucleotides can be used as a tool to study the effect of transiently silencing a certain gene, to bypass early lethality. An interesting example of such an approach has been described for studying the role of (pro)thrombin in Sickle cell disease (SCD) [46]. Genetically modified mice missing prothrombin are not viable due to fatal prenatal bleeding complications, so the authors were forced to choose another approach. Using prothrombin-specific designed gapmers (a class of ASOs where the internal sequence block is protected from nuclease degradation by artificially modified ribonucleotide monomers) prothrombin was transiently silenced SCD mice. It was shown that in SCD mice reduced levels of prothrombin lowered inflammation and endothelial cell dysfunction, and improved multiple SCD-associated organ pathologies and overall survival. These data imply that targeting a single coagulation factor i.e., prothrombin can ameliorate SCD pathology.

Using a comparable approach, the transcription factors hepatocyte nuclear factor 4α and CCAAT/enhancer-binding protein α were silenced using siRNAs, to study their regulatory role in the transcription of coagulation genes, demonstrating that by means of siRNAs the role of a gene in a certain process can be simply and rapidly unraveled [47].

## Oligonucleotides as therapeutics – animal studies

Besides using oligonucleotides as a tool for the study of gene function, they are also candidate therapeutics in the field of blood coagulation. All key proteins (with the exception of tissue factor and membrane bound receptors such as thrombomodulin, endothelial protein C receptor, and protease-activated receptors) are predominantly expressed in the liver. Moreover, a major advantage of using oligonucleotide drugs over other more conventional therapeutic strategies lies in the drugs’ target: (pre)mRNAs. Targeting proteins is complex due to their three-dimensional structure and small conformational differences which can cause complete loss of function of a drug, while (pre)mRNAs just differ in their gene-specific sequence. This means that conventional (“protein-based”) drugs have to undergo an elaborate screening process for compounds that inhibit the protein of interest in both experimental animals (during preclinical studies) and humans (actual trials), while for RNA-based drugs solely the oligonucleotide sequence needs to be adjusted. Moreover, oligonucleotide drugs can stably repress protein levels over the course of several weeks. In addition, oligonucleotides are not expected to become subject to an immunogenic response, inhibitors, or resistance during therapy.

The fundamental process of blood coagulation and the disease of venous thrombosis can be effectively inhibited in humans by using agents such as vitamin K antagonists, heparins, and the new generation of direct oral anticoagulants [48, 49]. All these therapeutics target components of the common pathway of coagulation. Outside the common pathway, coagulation factor IX might be an interesting candidate to target for the prevention of thrombosis. With an siRNA against this coagulation factor, it has been shown in rats that protein activity can be reduced with 50-99%. This reduced activity prevented experimental thrombosis without causing a bleeding phenotype. Over 99% inhibition of factor IX activity resulted in a bleeding phenotype, similar to factor IX deficient rats [50]. This observation makes an “intermediate” dose of siRNAs against factor IX (resulting in 50–99% reduction of activity) potentially interesting for therapeutic purposes.

Although targeting individual components from the coagulation cascade proved effective in preventing thrombosis, bleeding often remains a clinically relevant side effect. To circumvent this problem, coagulation factors XI and XII, both involved in the intrinsic, or contact-activation, pathway of coagulation have been introduced to target for preventing venous thrombosis [51–53]. Based on data derived from mouse studies, deficiency or transient lowering of factor XI and XII prevents thrombosis without (severe) bleeding as a side effect, unlike any other traditional coagulation factor. These reports coincide with human data, where individuals with a factor XII deficiency do not have a bleeding tendency, and factor XI deficient patients (although occasionally suffering from mild and injury-related events, hemophilia C) even seem to be protected from developing thrombotic events [54–56]. Moreover, both plasma proteins are exclusively produced in the liver, making them accessible for oligonucleotide therapy.

Coagulation factor XII is a plasma protease which upon activation initiates the intrinsic pathway of coagulation. When rats deficient in factor XII were compared with rats treated with a specific siRNA against factor XII (99% reduction in plasma factor XII), both showed protection in arterial as well as in venous thrombosis models [57]. In rabbits, a factor XII-specific ASO prolonged the time to occlusion in a catheter thrombosis model by 2.2 fold [58]. Comparable thromboprotective effects were obtained in mouse studies. Specific lowering of factor XII or (pre)kallikrein (an activator of the zymogen factor XII) using an ASO, resulted in reduced thrombus formation models for arterial and venous thrombosis, without a significant effect on normal hemostasis [59].

Coagulation factor XII is not only involved in (experimental) hemostasis, but also in release of bradykinin from high molecular weight kininogen, an inflammatory component mediating the dilation of blood vessels. Specific gain-of-function mutations in factor XII causing increased contact-mediated auto activation, which causes enhanced release of bradykinin and massive swelling, underlies hereditary angioedema type III [60]. Interestingly, experimental data in mice show that upon lowering factor XII or (pre)kallikrein using a specific oligonucleotide, certain characteristics associated with this disease can be prevented, which opens perspectives for treatment [61–63].

Coagulation factor XI is activated by factor XII towards activated factor XI. In mice, ASO-mediated specific and dose-dependent reduction of plasma factor XI showed an antithrombotic effect in various arterial and venous thrombosis models [40]. In parallel groups, mice were treated with either warfarin or enoxaparin, a commonly used vitamin K antagonist and low molecular weight heparin, respectively. Although the antithrombotic effect was considered equally effective in all groups, factor XI ASO-treated mice performed significantly better in a hepatectomy surgical bleeding model. In rabbits, lowering of factor XI prolonged the time to occlusion in a catheter thrombosis model, in a similar fashion as factor XII [58]. After these promising results, the role of factor XI and its therapeutic antithrombotic potential was tested in non-human primates. Again using mRNA-specific ASOs, it was first shown that plasma factor XI can be lowered in a dose-dependent manner without increasing the risk of bleeding [64]. In a second study, the antithrombotic potential of FXI-ASO was tested in non-human primates. In an arteriovenous shunt thrombosis model protection from thrombosis was reported, which coincided with a decreased thrombin generation [65]. These studies formed solid proof to continue with using an ASO against factor XI in clinical trials. Interestingly, silencing coagulation factors XI and XII also attenuated atherothrombosis in mice [66, 67], which implies that silencing these factors also might be useful in treating atherothrombotic complications.

Besides targeting procoagulant proteins to prevent venous thrombosis, targeting anticoagulants might also be useful in preventing disease, in a situation where the hemostatic balance has shifted towards bleeding i.e., hemophilia. Current therapies to treat hemophilia are based on administering plasma derived or recombinant proteins to replace the missing coagulation factors. The need for developing alternative treatment strategies is high, since a large portion of treated hemophilia patients develops inhibitory antibodies against these administered proteins. A promising approach which likely circumvents this problem is by silencing an anticoagulant using oligonucleotides, which can cause a shift towards a renewed hemostatic equilibrium. This hypothesis was tested by targeting antithrombin [68]. When factor VIII-deficient hemophilic mice were treated with an siRNA against antithrombin, the hemophilic phenotype disappeared in a dose-dependent manner without thrombotic complications. In the next animal model of choice, non-human primates (cynomolgus monkeys) with antibody induced FVIII deficiency, a dose-dependent silencing of antithrombin was again observed. Moreover, thrombin generation were restored to normal levels when treating the monkeys with the siRNA, which opened the perspective to test this drug in humans.

## Oligonucleotides as therapeutics – human studies

Recently, the first oligonucleotide drug targeting specifically the liver has been approved by the United States’ Food and Drug Administration. This therapeutic, an ASO called mipomersen (Kynamro®), directly targets the pre-mRNA of apolipoprotein B, which causes a significant downregulation of the corresponding plasma protein. This specific downregulation allows more release of very low density lipoprotein (VLDL) from the liver to the bloodstream, which will cause a drop of LDL-cholestorol levels in plasma [69, 70]. This is in line with the concept of the oligonucleotide drugs, which has been outlined previously. Mipomersen has been approved to treat homozygous familial hypercholesterolemia, and the availability of mipomersen is currently being investigated for more risk groups with high cholesterol levels to protect them from cardiovascular disease (http://www.fda.gov/NewsEvents/Newsroom/PressAnnouncements/ucm337195.htm).

The approval of mipomersen might cause a domino effect for the use of oligonucleotides which target mRNA in the liver for treating other human diseases: The first hurdle of safely delivering oligonucleotides to the liver has been overcome, so by using the same chemical formulation many more hepatic mRNAs can be targeted, which is an appealing prospect. Two oligonucleotide drugs are currently being tested in the field of coagulation, one for venous thrombosis treatment and one for hemophilia.

Coagulation factor XI has been investigated extensively in animal models for its role in coagulation, and whether it can be a therapeutic target to prevent venous thrombosis without introducing bleeding as a side effect [71]. Recently, the results of a clinical trial were reported, where an ASO against factor XI (FXI-ASO) was used for the prevention of deep-vein thrombosis after knee arthroplasty [72]. Previously, FXI-ASO was considered safe to use in humans since no side effects (including bleeding) were observed in a prior study [73]. When comparing a high and low dose FXI-ASO with conventional enoxaparin treatment, the bleeding risk was not significantly different (although less bleeding events were scored for FXI-ASO patients). Interestingly, treatment of patients with the low dose of FXI-ASO appeared safe considering the risk of deep-vein thrombosis, where the high dose was even considered superior to enoxaparin. Currently, a phase 2 clinical trial has been completed in which the efficacy of FXI-ASO in patients with end-stage renal disease was evaluated, with the primary goal to monitor the safety and tolerability of the ASO (ISIS 416858, Ionis pharmaceuticals). Significant and dose-dependent reductions in FXI activity were observed upon treatment (http://ir.ionispharma.com/phoenix.zhtml?c=222170&p=irol-newsArticle&ID=2217775).

As outlined previously, on the other side of the spectrum hemophilia can be treated by transient lowering of antithrombin, to restore the hemostatic balance. Currently, a human-specific antithrombin siRNA named *Fitusiran* is being tested in phase 1 clinical trials. This siRNA, amongst others candidate siRNAs which are tested in clinical trials, is chemically modified to improve its systemic stability and is conjugated to a GalNAc-ligand, which allows hepatocyte-specific uptake [17]. The phase 1 clinical trial is done in a diverse group of individuals, including healthy volunteers, patients with hemophilia A or B, and with or without inhibitors against supplemented recombinant proteins [74]. The goal of this trial is to evaluate the safety and tolerability of multiple doses of subcutaneously administered *Fitusiran*. Moreover, thrombin generation assays in plasma derived from hemophilia patients are performed to test whether they recover towards levels of healthy individuals. Preliminary data show that hemophilia patients did not develop any serious (thromboembolic) complications because of the drug. Considering functionality of the drug, when antithrombin levels were lowered to >75% thrombin generation peaks were increased in a dose-dependent manner with 289%. Positive interim results of two clinical trials (phase 1 and phase 2) for *Fiturisan* have been reported late 2016 (ALN-AT3SC, Alnylam pharmaceuticals, http://www.alnylam.com/capella/presentations/fitusiran-data-ash-2016/).

## Conclusions

Hepatic in vivo gene silencing using oligonucleotides has shown to be specific and rapidly applicable in multiple animal models. The approach can not only serve as an alternative for using genetically modified animals (although restricted to the liver), it can also help unraveling biological processes which cannot be studied otherwise. Moreover, promising preclinical trials for the development of new oligonucleotide drugs to treat thrombosis and hemophilia have been reported.

Since the introduction of mipomersen in 2013, the first oligonucleotide drugs are slowly finding their way towards the clinic (currently, three ASOs and no siRNAs yet). Together with all the preclinical data, it has been shown that the process of delivering oligonucleotides to the liver can be achieved in a safe way (thus far, no specific toxicities have been observed). Currently, preventing adverse events (at the injection site or from infusions) of oligonucleotide drugs remains an important issue, which means that reducing the dosage of the drugs is crucial. The dosages can be lowered by, for instance, the conjugation of GalNAc-ligands to oligonucleotides to improve cell-specific delivery [22]. When these delivery and dosage issues can be solved, more clinical situations will present where using oligonucleotides will be regarded advantageous over using classical therapies.

Thrombosis and hemophilia are two diseases where the current treatment can be improved, and the two drugs which are currently investigated in clinical trials show promise. An important advantage compared to current therapies for hemophilia is that the effect of oligonucleotides remains for a longer time period, in contrast to the low-interval dosing of conventional treatment. This would mean that patients will go from daily to monthly treatment. Moreover, hemophilia patients who develop inhibitors against supplemented factor VIII or IX will benefit from this novel treatment option, since oligonucleotides will likely not provoke this immune response.

Although the mode of action of oligonucleotides in humans compared to current therapies can be regarded as a great benefit, it can be a problem in situations when rapid reversal of the silenced protein is necessary. In some cases, singular treatment with recombinant proteins will be a solution, although correct dosing would be crucial. A more elegant solution would be to use an oligonucleotide antidote. Recently, it was shown in mice that upon prothrombin-specific ASO treatment, complementary sense oligonucleotide antidote (SOAs) reversed the antithrombotic phenotype by specifically binding of ASOs intracellularly [75]. Even though these data are promising, therapeutic usefulness has to be proven for individual ASOs. Moreover, it has been shown that reversal of siRNA therapy is feasible using complementary high affinity oligonucleotides as a “synthetic target” or decoy to abrogate silencing activity of antisense-loaded RISC (Reversir™, http://www.alnylam.com/web/assets/Reversir_OTS_101315.pdf). Currently, the strategy has been reported to be functional for reversal of the effect of an siRNA against coagulation factor IX. The major disadvantage of this oligonucleotide-specific approach is that it may take too long for the antidote to restore original levels of the initially silenced protein. For this reason, in the case of a sudden event such as a surgical intervention, an oligonucleotide-specific antidote may not be adequate and recombinant proteins or fresh frozen plasma treatment are still necessary.

In conclusion, in fundamental research oligonucleotides targeting coagulation factors in the liver can serve multiple purposes, and can improve our understanding about coagulation biology, in physiology and disease. Considering oligonucleotide-based silencing of coagulation factors to treat thrombosis or hemophilia, for the short term, validation studies focused on the safety and efficacy of the products for clinical applications look promising. For the long term, a whole new class of drugs might enter the market, where oligonucleotides might have the potency to be delivered to other organs than the liver, or allow allele-specific silencing. Moreover, an entire panel of genes could be silenced by including multiple oligonucleotides in a single treatment. Time will tell whether the new generation of RNA-targeting therapeutics can fulfill its promise.
